# Complete Genome Sequences of *Mycobacterium smegmatis* Phages Dove and Issimir

**DOI:** 10.17912/micropub.biology.001432

**Published:** 2025-01-16

**Authors:** Victoria Frost, Israel Bellinger, Riley Burn, Chastity Chisholm, Fisher Cobb, Hannah Duncan, Kalli Green, Madelynn Harding, Amari Johnson, Rachel Leek, Breanna Menard, Ciaran Murphy, Destiny Thompson, Gwendolyn Tomlin, Kristi Westover

**Affiliations:** 1 Department of Biology, Winthrop University, Rock Hill, South Carolina, United States

## Abstract

We announce the discovery of two mycobacteriophages isolated from soil in Rock Hill, South Carolina. Phage Dove has a genome sequence length of 108,976bp, a siphovirus morphology, and a predicted temperate lifecycle. Phage Issimir has a genome sequence length of 155,564bp, a myovirus morphology, and a predicted lytic lifecycle.

**Figure 1. Particle and plaque morphologies for Dove and Issimir:**
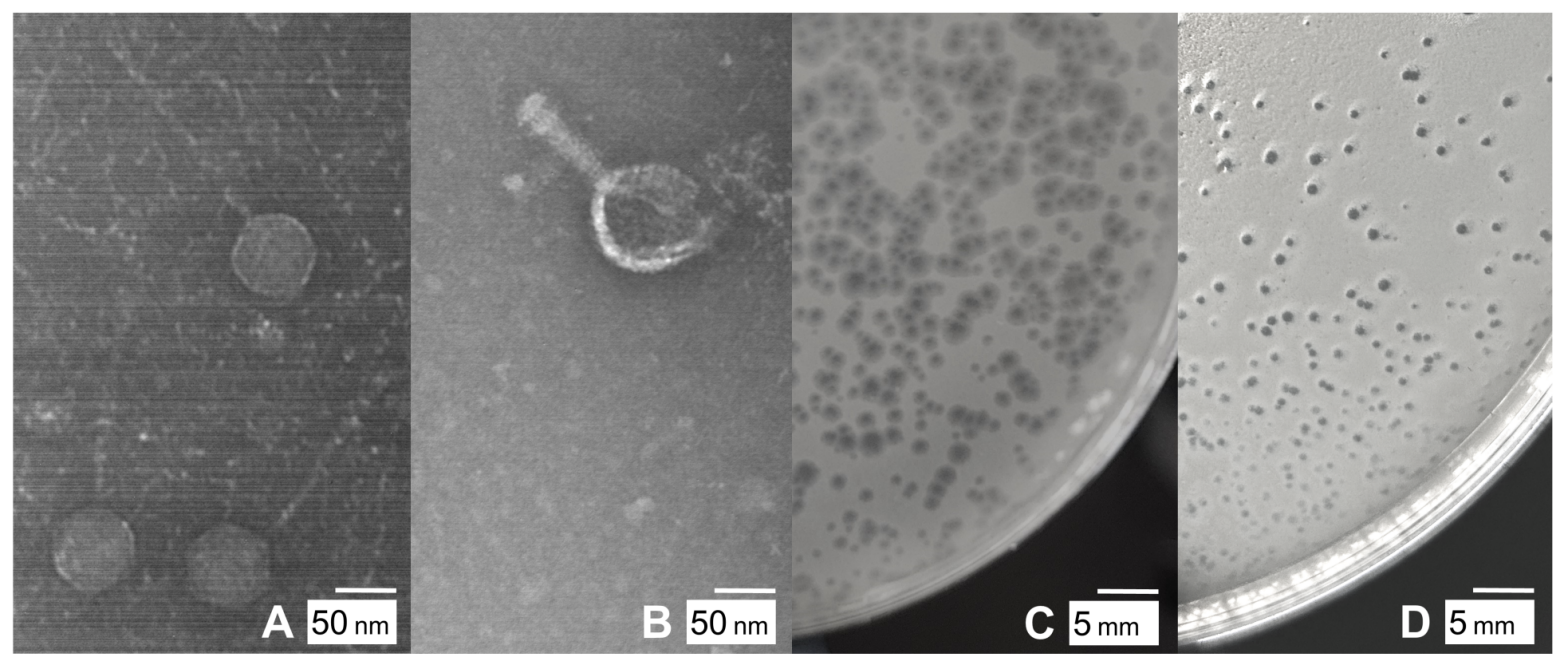
Transmission electron micrographs of Mycobacterium phages Dove (A) and Issimir (B). Phage lysates were negatively stained with 1% uranyl acetate, and images taken with JEOL JEM-1230 TEM at 100kV acceleration voltage. Dove (C) forms plaques with a clear center surrounded by a turbid edge. Issimir (D) forms clear plaques.

## Description

Continued isolation and characterization of bacteriophages is important to help mitigate the increasing global burden of antibiotic resistant bacterial infections (Antimicrobial Resistance Collaborators 2022; Chinemerem Nwobodo et al., 2022). Therapeutic phage use has demonstrated positive outcomes in a number of recent clinical trials including patients with gastrointestinal (Stellfox et al., 2024) and pulmonary infections (Nick et al., 2022) . Here, phages Dove and Issimir were discovered in moist soil collected under bushes on Winthrop University’s campus, Rock Hill, SC (see Table 1 for GPS coordinates). Microbes were loosened from particulate matter by shaking (2hr) in 7H9 broth containing 1 mM CaCl _2_ , using standard procedures (Zorawik et al., 2024) , and following centrifugation (2,000 x g, 10 min) the supernatant was filtered (0.22 µm pore size). A fraction of each filtrate was inoculated with *Mycobacterium smegmatis* mc ^2^ 155 and shaken at 250 rpm (37°C) for 2 to 4 days to enrich for mycobacteriophages. Standard plaque assays were performed to assess each sample for the presence of mycobacteriophage. Briefly, samples were mixed with molten 7H9 soft agar and *M. smegmatis* mc ^2^ 155, then plated on 7H9 agar plates. Plates were incubated at 37°C. Dove was isolated from unenriched filtrate and produced small (1-2mm diameter) clear plaques with a turbid edge. Issimir was isolated from an enriched filtrate and produced very small (0.5–1mm diameter) clear plaques. Transmission electron microscopy of phage lysates showed Dove has a long, non-contractile tail (a siphovirus morphology), while Issimir has a short contractile tail (a myovirus morphology).

Phage DNA was extracted from lysates using the Wizard DNA clean-up kit (Promega). Sequencing libraries were constructed using the NEBNext Ultra II FS DNA library prep kit and genomes were sequenced (Illumina MiSeq v3 platform). Sequence reads (150-bp single-end raw reads) were assembled using Newbler v2.9. Consed v29 was used to check for accuracy and coverage and to determine genomic termini (Miller et al., 1020; Gordon and Green, 2013) . Sequencing results and phage genome characteristics are presented in Table 1, which includes the predicted number of genes, and phage cluster designation based on gene content similarity (GCS) of at least 35% to phages within the Actinobacteriophage database ( https://phagesdb.org/ ) (Russell and Hatfull 2017; Pope et al., 2017) .

Genome sequences were annotated using PECAAN (v20240320) (Rinehart et al., 2016) embedded with Glimmer v3.02 (Delcher et al., 2007) , GeneMark v4.28 (Besemer and Borodovsky 2005) , Starterator v558 ( http://phages.wustl.edu/starterator/ ), Phamerator v3 (Cresawn et al., 2011) , HHPred v3 (Söding et al., 2005), TMHMM v1.0.24 (Hallgren et al., 2022) , and BLASTp v2.13.0 (Altschul et al., 1990) . The non-redundant and actinobacteriophage databases v581 were used for homology searches with BLASTp v2.13.0 (Altschul et al., 1990) . Databases PDBmmCIF70, Pfam-A v37, SCOPe v2.08, and NCBI Conserved Domain were used for homology searches with HHPred v3 (Söding et al., 2005). Transfer RNAs were identified using Aragorn v1.1 integrated into DNA Master (Pope and Jacobs-Sera 2018) , Aragorn v1.2.38 (Laslett and Canback 2004) , and tRNAscan-SE v2.0.6 (Lowe and Eddy 1997) . Putative transmembrane domains were assessed using TMHMM v2 (Hallgren et al., 2022) , SOSUI v1.11 (Hirokawa et al., 1998) , and TOPCONS v2 (Bernsel et al., 2009) . Default parameters were used for all bioinformatic analyses unless otherwise stated.

Following annotation, approximately 26% of the predicted genes in both genomes could be assigned possible functions (63 genes in Dove, and 58 genes in Issimir). These include protein-coding genes for the tail, capsid, lysin A, lysin B, holin and glycosyltransferases. Genes encoding an immunity repressor and tyrosine integrase were identified in Dove, suggesting Dove is a temperate phage consistent with experimental evidence of lysogen formation for other cluster J phages (Pope et al., 2013) . Dove lacks identifiable introns previously observed in a small subset of cluster J phages (Pope et al., 2013) . As is typical of C1 phages, Issimir’s genome encodes a ThyX-like thymidylate synthase, many tRNAs (37), and no identifiable immunity repressor or integrase functions. Specifically lacking is the immunity repressor of cluster A mycobacteriophages that is found in a small subset of C1 phages and thought to be maintained for superinfection immunity (Jain and Hatfull 2000; Pope et al., 2011) .

**Data availability**

The complete genome sequences of phages Dove and Issimir are available in GenBank (accession no. PQ184790 and PQ184821, respectively). The raw sequencing reads are available in the NCBI SRA under accession no. SRX25999121 and SRX25999122 respectively. The Actinobacteriophage sequencing BioProject accession number is PRJNA488469.

**TABLE 1** Phage sample locations and genome assembly results

**Table:** 

Phage Name	Dove	Issimir
Sample Location (GPS)	34.937476N, 81.03085 W	34.937476N, 81.03085 W
Sequencing Reads	154K	1M
Sequencing coverage (X)	212	991
Genome size (bp)	108,976	155,564
Genome ends	3’ single stranded overhang (5’- ATCC -3’)	Circularly permuted
GC content (%)	60.7	64.8
No. of genes	230	231
Cluster	J	C1
